# Trends in mortality from pneumonia in the Europe union: a temporal analysis of the European detailed mortality database between 2001 and 2014

**DOI:** 10.1186/s12931-018-0781-4

**Published:** 2018-05-04

**Authors:** Dominic C. Marshall, Ross J. Goodson, Yiwang Xu, Matthieu Komorowski, Joseph Shalhoub, Mahiben Maruthappu, Justin D. Salciccioli

**Affiliations:** 1 Oxford University Clinical Academic Graduate School, John Radcliffe Hospital, Oxford, UK; 2 Department of Medicine, Imperial College London, London, UK; 3 School of Clinical Medicine, University of Cambridge, Cambridge, UK; 4 Department of Surgery and Cancer, Faculty of Medicine, Imperial College London, London, UK; 5 Academic Section of Vascular Surgery, Department of Surgery and Cancer, Imperial College London, London, UK; 6 Cera Care, London, UK; 7 Department of Medicine, Mount Auburn Hospital, Cambridge, MA USA

## Abstract

**Background:**

Pneumonia is responsible for approximately 230,000 deaths in Europe, annually. Comprehensive and comparable reports on pneumonia mortality trends across the European Union (EU) are lacking.

**Methods:**

A temporal analysis of national mortality statistics to compare trends in pneumonia age-standardised death rates (ASDR) of EU countries between 2001 and 2014 was performed. International Classification of Diseases version 10 (ICD-10) codes were used to extract data from the World Health Organisation European Detailed Mortality Database and trends were analysed using Joinpoint regression.

**Results:**

Median pneumonia mortality across the EU for the last recorded observation was 19.8 / 100,000 and 6.9 / 100,000 for males and females, respectively. Mortality was higher in males across all EU countries, most notably in Estonia and Lithuania where the ratio of male to female ASDR was 4.0 and 3.7, respectively. Gender mortality differences were lowest in the UK and Demark with ASDR ratios of 1.1 and 1.5, respectively. Pneumonia mortality across all countries decreased by a median of 31.0% over the observation period. Countries that demonstrated an increase in pneumonia mortality were Poland (males + 33.1%, females + 10.2%), and Lithuania (males + 6.0%).

**Conclusions:**

Mortality from pneumonia is improving in most EU countries, however substantial variation in trends remains between countries and between genders.

**Electronic supplementary material:**

The online version of this article (10.1186/s12931-018-0781-4) contains supplementary material, which is available to authorized users.

## Background

Pneumonia, together with other lower respiratory tract infections (LRTI), is the fourth leading cause of death worldwide with approximately 2.38 million deaths attributed to LRTI in 2016 [1, 2]. In the European Union (EU), pneumonia remains the most frequent cause of death from infection, especially in the elderly and those with co-morbidities [3–7]. In 2014 pneumonia was responsible for 118,300 deaths across the EU [8] and a previous review reports an annual incidence of 1.08–1.7 per 1000 population in Europe [9]. An increase in pneumonia hospital admissions, costs and potentially deaths is likely given the ageing European population [10, 11]. Historic data indicates that pneumonia costs approximately 10.1 billion Euros annually with likely additionally indirect costs from time off work [6]. Pneumonia can be a primary disease process, or may complicate and develop secondary to another disease such as dementia, frailty or in immunocompromised patients. It is therefore important to be able to differentiate potentially preventable pneumonia-related deaths from intractable ones, often occurring as a consequence of the individual’s co-morbidities. From an epidemiological point of view, such differentiation might reflect overall management of potentially preventable deaths, or disease prevention in the first instance.

Despite overall low rates in Europe, significant variation in mortality from pneumonia exists amongst countries and between geographic regions [10, 12]. The determinants of such differences are multifaceted: differences in smoking regulations, healthcare system setup, pneumococcal vaccination, or antibiotic resistance and regulations to control air pollution in a region may contribute to fluctuations in pneumonia incidences and mortality trends [13, 14]. The mortality variability also likely reflects variation in underling incidence of pneumonia within a population. Burden of co-morbidities such as COPD and HIV have been demonstrated to be associated with increase incidence of community acquired pneumonia in addition to lifestyle factors such as smoking and high alcohol intake [9]. An analysis of the global burden of disease indicates that mortality from LRTI is decreasing globally [15] and a previous report has highlighted that pneumonia mortality has decreased by 3.8% per year between 1999 and 2013 in the United States [16]. To date, there has been no published comparison of pneumonia mortality trends across Europe using the tenth revision of the International Statistical Classification of Diseases and Related Health Problems (ICD) version 10 medical classification system.

Our primary objective was to describe patterns of pneumonia mortality using available data from EU countries between 2001 and 2014. Our primary hypothesis was that there would be an observable decline in pneumonia-related mortality across EU states. To test this hypothesis, we performed a temporal analysis of the World Health Organisation (WHO) European Detailed Mortality Database (EDMD) [17] and used Joinpoint regression analysis to identify significant changes in trends during this time-period.

## Methods

### Study design and data source

For this temporal analysis of national mortality statistics, mortality data for pneumonia, as coded by the ICD-10 system, from individual states within the EU were derived from the EDMD for the years 2001–2014. The EDMD data is compiled from the larger WHO mortality database. The WHO mortality database is produced from national vital registration systems where the underlying cause of death is coded by the appropriate national authority. The WHO analyse the quality and completeness of these data and provide reports of these analyses [18]. This approach has been used previously for similar analyses [19, 20]. Mortality data was standardised to the European Standard Population [21]. We included countries for analysis if they were member states of the European Union and had a population greater than 1 million. We excluded any countries which were missing > 20% of data (i.e. greater than any 3 years of missing data). The EDMD is collated from data submitted to the WHO by European member states, data submitted includes cause of death, age of death, sex of individual. Separate data on population size, numbers of males and females and age category is also collected.

The EDMD uses both ICD-9 and ICD-10 to categorize causes of death. For the purposes of the current investigation, we chose a priori to analyse recent trends in mortality using only the ICD-10 classification, as the introduction of ICD-10 changed the definition of pneumonia substantially so that if pneumonia was the end-point in a chronic disease process then the disease, instead of the pneumonia, would be recorded as the underlying cause of death. We abstracted from the database ICD-10 codes J12–18 for pneumonia, excluding influenza-related pneumonia (J09, J10, J11), aspirational pneumonia (J69, O74, O29, O89, P24.9) and congenital pneumonia (P23, P24.9, P28.9).

The majority of EU member states have an estimated level of coverage of deaths that are registered with cause of death data of > 95% for the time periods included in this study; the exceptions are Cyprus (> 78.2%) and Croatia (> 94.9%). This rate is calculated by dividing actual reporting by estimated mortality rate. Population and birth recording in all countries exceeds 90%, as per the WHO standard for inclusion in the database.

### Data handling and statistical analysis

Country-specific and sex-specific mortality data were abstracted from the WHO EDMD (url: http://data.euro.who.int/dmdb/; date of access: February 2017) and collated into Microsoft Excel spreadsheets. Mortality data are expressed as age-standardised death rates (ASDR) per 100,000 population using European Standard Population. Three-year average ASDR were calculated for the start (2001–2003) and end (2012–2014) of the observation period to allow comparison of mortality rates over the study period. Countries were ranked based on 3-year average mortality at the start and end of the observation period, and also for percentage change across the observation period. Where countries had incomplete data at the start or end of the observation period, 2-year averages were used and, in one instance (Denmark), the last year of available data was used.

Statistical trends were assessed using Joinpoint software (Version 4.1.1.1) provided by the United States National Cancer Institute Surveillance Research Program [22]. For the purpose of Joinpoint analysis, imputed data was used in a last observation carried forward manner. Joinpoint regression analysis assesses changes in linear slope for mortality trends over time [23] as described previously [24]. Briefly, it assesses the overall trends in mortality and identifies the best-fitting points where mortality rates change significantly (significant increase or significant decrease). The analysis initially starts with no Joinpoints and tests for significant changes in the model with sequential addition of points where there is significant change in the slope of the line. Each joinpoint in the final model indicates a statistically significant change in mortality trend and Joinpoint software computes the Annual Percentage Change (APC) for each piecewise trend by means of a generalised linear model, assuming Poisson distribution.

### Post-hoc analysis

Previous reports have demonstrated worse health outcomes in former communist countries who joined the EU after 2004 compared with those that joined early [20]. Countries who joined the EU prior to 2004 were Austria, Belgium, Denmark, Finland, France, Germany, Netherlands, Spain, Sweden and the United Kingdom (UK). Countries who joined the EU post-2004 were Croatia, Czech Republic, Estonia, Hungary, Latvia, Lithuania, Poland, Romania and Slovakia. We performed a post-hoc analysis to assess whether the observed differences in gender-specific mortality followed this pattern. First, we compared the raw differences and ratios in male and female ASDR, for the time period 2012–2014, for countries who joined pre- and post-2004 using the Wilcoxon-Rank sum test. We then constructed linear mixed-models to compare the ASDR ratio between the two groups of European countries. For this analysis, we treated ASDR ratio as the outcome and category of European country (early vs late to join EU) as the primary exposure of interest. We checked the interaction between country category and year of observation, and retained the interaction in the model if *p* < 0.05. For linear models, we assessed model structures including compound symmetry, auto-regressive, and unstructured. We used Akaike information criterion (AIC) for final model selection.

In addition to Joinpoint software, data was collated into Microsoft Excel and analysed using SAS (Version 9.4) and R Studio (Version 1.1.383).

### Sensitivity analysis

We performed a single sensitivity analysis to assess contributions of influenza virus on trends in pneumonia-related mortality. We chose a priori to exclude from the analysis deaths which were recorded to be secondary to influenza as these are likely to represent a distinct pathological phenomenon with high annual variations in case fatality. However, we chose to perform a single sensitivity analysis which included influenza-related mortality to assess for significant contributions to overall trends. For this analysis, we included cases related to ICD-10 definitions J09, J10, and J11 in addition to cases for J12 – J18. We repeated the Joinpoint analysis, as described above and the results are reported in the Additional file 1.

## Results

A total of 19 EU countries had sufficient data for analysis for the chosen time-period; 12 countries had complete data from 2001 to 2014, and 7 countries were missing 1 or 2 years of data. Malta and Luxembourg were excluded from analysis due to populations fewer than 1 million citizens. Bulgaria, Cyprus, Italy, Ireland, Greece, Portugal and Slovenia were excluded due to > 20% (> 3 years) missing data. Austria was a late adopter of ICD-10 (2002).

**Fig. 1:**
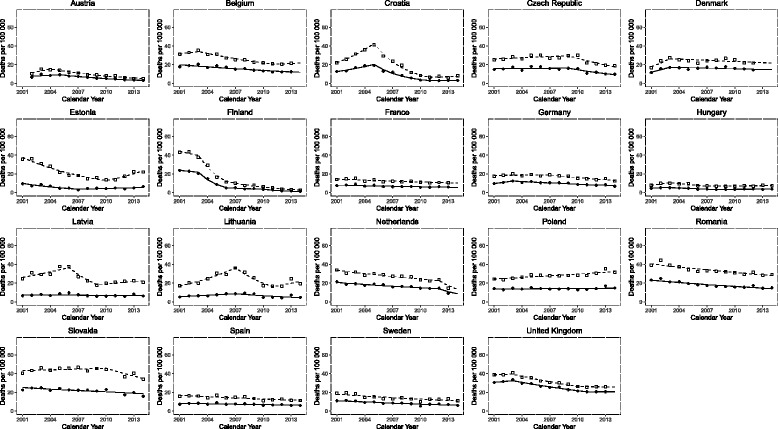
Pneumonia mortality trends of male and females in 19 European countries. Lines represent result of Joinpoint analyses: dashed and continuous lines for males and females, respectively. Squares (males) and circles (females) represent raw data; where symbol is absent, data has been imputed for the respective year

Median mortality across the EU for last recorded observation was for males and females, respectively, 19.8 / 100,000 (IQR 11.3–21.8) and 6.9 / 100,000 (IQR 5.3–13.4). Table 1 summarises the first and last three-year average mortality, i.e. 2001–2003 and 2012–2014 (unless otherwise specified), of males and females for each of the 19 countries included in the study. Figure 1 demonstrates individual data points for each country, sex and year. For males, the countries with the highest mortality rates were Slovakia (37.0 / 100,000), Poland (32.5 / 100,000) and Romania (29.8 / 100,000). The highest mortality rates for females were identified in the UK (20.6 / 100,000), Slovakia (17.6 / 100,000) and Romania (15.7 / 100,000). The lowest mortality rates for pneumonia in males were demonstrated in Finland (3.3 / 100,000), Austria (5.5 / 100,000) and Croatia (7.2 / 100,000). For females, the lowest rates were in Finland (1.6 / 100,000), Croatia (3.2 / 1,000,000) and Austria (3.6 / 100,000).

**Table 1:** The first and last three-year averages and percentage change in age standardised mortality rate for pneumonia in 19 European countries during 2001–2014. The start represents the average of 2001–2003, and the end represents the average of 2012–2014, unless otherwise specified. Rank for 2001–2003, 2011–2013 and % change is reported

Country	Male			Female		
	Start	End	% Change	Start	End	% Change
Austria*	12.9(2)	5.50(2)	−57.4(3)	8.33(6)	3.55(3)	−57.41(3)
Belgium*	33.27(14)	21.09(13)	−36.61(6)	19.15(14)	12.45(14)	−35.01(8)
Croatia	26.48(11)	7.24(3)	−72.65(2)	14.31(11)	3.15(2)	−78.01(2)
Czech Republic	26.45(10)	19.80(10)	−25.13(13)	15.99(13)	10.67(12)	−33.31(9)
Denmark*	22.88(8)	21.78(15)	−4.8(17)	14.81(12)	14.39(15)	−2.82(18)
Estonia	34.39(15)	20.61(12)	−40.07(5)	8.44(7)	5.19(5)	−38.45(5)
Finland	41.56(18)	3.26(1)	−92.16(1)	22.34(16)	1.61(1)	−92.79(1)
France*	14.77(3)	10.82(5)	−26.73(11)	8.20(5)	6.21(7)	−24.27(14)
Germany	18.83(5)	13.79(8)	−26.77(10)	11.00(8)	7.72(11)	−29.81(11)
Hungary	9.34(1)	7.46(4)	−20.19(15)	5.08(1)	3.72(4)	−26.75(12)
Latvia	28.27(12)	21.75(14)	−23.07(14)	7.15(3)	6.77(9)	−5.2(17)
Lithuania	19.16(7)	20.30(11)	5.95(18)	6.01(2)	5.49(6)	−8.63(16)
Netherlands*	32.12(13)	19.20(9)	−40.22(4)	19.82(15)	11.94(13)	−39.75(4)
Poland	24.44(9)	32.53(18)	33.1(19)	13.87(10)	15.28(16)	10.14(19)
Romania	40.95(17)	29.77(17)	−27.3(9)	23.19(17)	15.67(17)	−32.44(10)
Slovakia	43.29(19)	37.04(19)	−14.44(16)	24.01(18)	17.61(18)	−26.65(13)
Spain	16.02(4)	11.86(6)	−25.96(12)	7.98(4)	6.29(8)	−21.25(15)
Sweden	19.03(6)	12.37(7)	−34.97(7)	11.10(9)	6.92(10)	−37.68(6)
United Kingdom*	39.58(16)	25.90(16)	−34.55(8)	31.89(19)	20.64(19)	−35.26(7)

*For Austria, 2002–2003 were used for start of period with % change 2002–2003 vs 2012–2014. For Belgium, 2012–2013 were used for end of period with % change 2001–2001 vs 2012–2013. For Denmark, 2012 was used for end of period with % change 2001–2003 vs 2012. For France, 2012–2013 were used for end of period with % change 2001–2003 vs 2012–2013. For Netherlands, 2012–2013 were used with % change 2001–2003 vs 2012–2013. For the UK, 2012–2013 were used for end of period with % change 2001–2003 vs 2012–2013

**Fig. 2:**
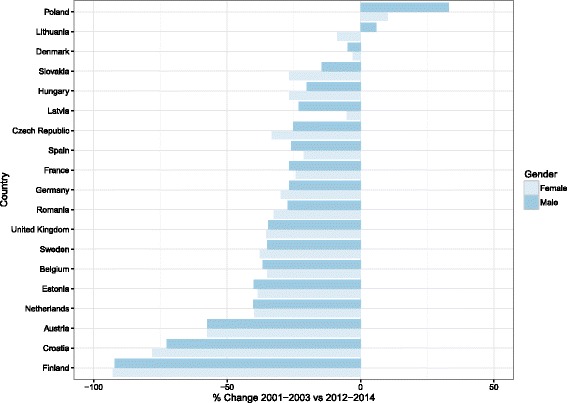
Ranked percentage change data comparing average of European age standardised death rate for 2001–2003 vs 2012–2014, unless otherwise specified. For Austria, 2002–2003 were used for start of period with % change 2002–2003 vs 2012–2014. For Belgium, 2012–2013 were used for end of period with % change 2001–2001 vs 2012–2013. For Cyprus, 2004 was used for start of period, and 2012–2013 were used for end of period with % change 2004 vs 2012–2013. For Denmark, 2012 was used for end of period with % change 2001–2003 vs 2012. For France, 2012–2013 were used for end of period with % change 2001–2003 vs 2012–2013. For Netherlands, 2012–2013 was used with % change 2001–2003 vs 2012–2013. For Portugal, 2002–2003 were used for start of period with % change 2002–2003 vs 2012–2014. For Slovenia, 2010 was used for end of period with % change 2001–2003 vs 2010. For the UK, 2012–2013 were used for end of period with % change 2001–2003 vs 2012–2013

The percentage change between the start and end of our observation period was calculated and is shown in Fig. 2. The majority of the 19 countries demonstrated a decrease in pneumonia-related mortality for both males and females over the study period. Exceptions to this included Poland (+ 33.1% and + 10.2%, for males and females, respectively) and Lithuania (+ 6.0% for males) (Table 1). In addition, pneumonia mortality across all countries declined by a median value of - 31.0% (IQR: -21.7% - -38.3%) for males and − 32.4% (IQR: -22.8% – -38.0%) for females, with Finland having the largest decline of -92.2% for males and -92.8% for females.

The trends in pneumonia mortality in individual countries between 2001 and 2014 were also analysed using Joinpoint regression [23] (Fig. 1). ASDR declined continuously in France, Romania, Spain and Sweden, with an estimated annual percentage change (EAPC) of -2.7% / -2.7%, -2.8% / -3.7%, -2.9% / -2.2%, -3.8% / -4.5%, for males and females, respectively. The sharpest decrease observed was in Croatia for the period 2006–2010 where an EAPC of -26.8% and -27.4% was modelled for males and females, respectively. Croatia also had the steepest increase at the start of the observation period with an EAPC of + 17.8% and + 12.6% for males and females, respectively, between 2001 and 2004. Several countries, including Austria, Belgium, Croatia, Czech Republic, Denmark, Germany, Hungary, Latvia, Lithuania, and Slovakia, demonstrated an initial increasing trend in the rate of pneumonia mortality before rates declined towards the mid-to-end of the observation period.

**Fig. 3:**
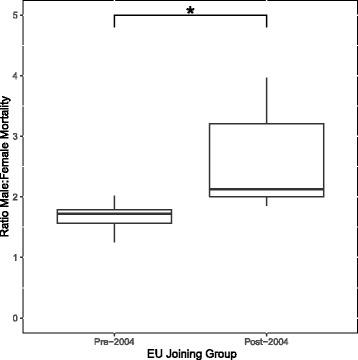
Ratios of male to female mortality for the period 2012–2014. The pre-2004 joining group was composed of Austria, Belgium, Denmark, Finland, France, Germany, Netherlands, Spain, Sweden and the UK. The post-2004 joining group was composed of Croatia, Czech Republic, Estonia, Hungary, Latvia, Lithuania, Poland, Romania and Slovakia. * *p* < 0.001

Pneumonia mortality was higher in males across all EU countries, most notably in Estonia and Lithuania where the ratio of average male to female ASDR was 4.0 (20.6 / 5.2 per 100,000) and 3.7 (20.3 / 5.5 per 100,000), respectively, at the end of the study period. In contrast, countries such as the UK and Denmark showed a less profound difference between male and female pneumonia ASDR, with male to female ratios of 1.1 (6.3 / 5.6 per 100,000) and 1.5 (21.8 / 14.4 per 100,000), respectively. Ratios for male to female mortality are represented in Fig. 3. For the period 2012–2014, the male to female mortality ratio was significantly lower for countries the joined the EU prior to 2004 compared with those that joined after (*p* < 0.001). The male to female mortality ratios for countries that joined the EU prior to 2004 remained relatively stable from 1.71 (SD 0.25) in 2001 to 1.75 (SD 0.25) in 2014. This compares to countries that joined the EU post-2004 which demonstrated a similarly stable, albeit greater, male to female mortality ratios from 2.34 (SD 0.91) in 2001 to 2.36 (SD 0.92) in 2014. The linear mixed model confirmed a significant difference between the two country categories (*p* = 0.03).

The sensitivity analysis to assess the overall contribution of influenza deaths revealed no significant differences in overall pneumonia-mortality trends over the observation period. A figure to compare trends with and without influenza cases is provided as Additional file 1: Figure S1.

## Discussion

This observational study of World Health Organisation European Detailed Mortality Database age-standardised mortality data found a low rate of pneumonia death across the EU, but did highlight considerable variability between the included countries. From years 2001 to 2014, there was a moderate decline in rates of death from pneumonia in the majority of European countries. This decrease is not universal, with specific exceptions in Lithuania and Poland. Furthermore, we identified a disparity in male and female mortality rates from pneumonia in the majority of countries assessed.

Reporting the trends in pneumonia mortality in European countries is important to allow comparison of statistical data at an international level, to assess the effectiveness of public health measures, and support the implementation of future interventions and initiatives. Our study aim was to report pneumonia mortality data from all countries of the EU that are classifying the disease using a single standardised international coding system. Time since the implementation of the ICD-10 mortality coding system is reaching 15 years in the majority of EU countries. To our knowledge, this study is the first to analyse pneumonia mortality trends across this study period using this system.

Unfortunately, incidence data for pneumonia mortality across Europe is severely lacking with only the UK, Finland and Spain having produced any precise population data. It is known that pneumonia mortality varies substantially between different European countries [25], but comparison is difficult due to differences in data acquisition and reporting, hospital admission criteria, and differences in disease management. Further difficulties arise from the lack of a universally recognised definition that can be used to reliably diagnose pneumonia [26–28]. The absence of a productive cough, administration of pre-diagnosis antibiotics, and the absence of a definitive diagnostic microbiological test can also impair the quality and reliability of pneumonia mortality data [7, 26].

Due to difficulties in clinical diagnosis, ICD codes are used to identify diseases and has become an international standard for classification. This study utilises the ICD-10 coding system, which allows the identification of death caused by a pneumonia infection and not as an end-point of a different underlying disease process. Studies have validated the use of ICD-10 codes to retrospectively identify pneumonia mortality and suggest its superiority to both the previous ICD-9 system and the use of clinical signs, symptoms and radiology [29].

Aggressive smoking cessation campaigns, the uptake of pneumococcal and influenza vaccination strategies, more widespread regulated use of guideline antibiotics [30–32], as well as other advances in medical care are expected to lead to a progressive and steady decline in pneumonia mortality, similarly to that which has been observed in, for example, sepsis [33], asthma [34] or maternal mortality [35]. The overall trend observed in this study confirms this assumption, with a median decline in pneumonia mortality of approximately 31% between 2001 and 2014. However, our study highlights considerable national differences in mortality rates, with a greater than twelve-fold difference in mortality between the countries with the highest and lowest mortality rates (2012 to 2014 data). Additionally, pneumonia mortality increased across the study period in countries such as the Poland and Lithuania (males only).

Overall, there was a gender discrepancy with rates of pneumonia mortality higher in males than in females. This gender difference was higher throughout the observation period, typically in Eastern European countries who joined the EU after 2004. These are generally poorer with lower standards of living. The greater ratio of male to female mortality in Eastern compared with Western Europe may reflect higher rates of smoking in males [36], pre-disposing individuals to respiratory disease. This is supported by previous findings that smoking-attributable mortality is higher in Eastern Europe [37].

A rise in incidence and hospitalisation for pneumonia, especially in elderly individuals, has been documented across Europe [3, 7, 38, 39]. The high prevalence of comorbidities in these populations is well documented and has been linked to a poorer prognosis for pneumonia [5, 40]. A recent review of pneumonia mortality literature across Europe reported a number of variables that have been shown to be associated with pneumonia mortality, including age, the presence of a pleural effusion, polymicrobial pneumonia, underlying disease severity, failed initial therapy, pneumococcal infection, multilobar infection, recent hospitalisation, impaired mental states, and acute kidney injury [6]. Other explanations may include exposure to social, economic or environmental risk factors, lower standards of care and differences in thresholds for hospital admission (i.e. differences in whether sick patients are more or less likely to be admitted between countries). All these factors could explain why pneumonia mortality remained unchanged or increased in some countries during the period of interest.

Several factors may contribute to the future burden of pneumonia mortality in Europe. It is well established that pneumonia is predominantly a disease of the elderly and that mortality increases as quality of life decreases for patients with advancing age [6]. The aging population of Europe has been predicted to continue to rise and this may result in a reversal in the overall decline seen here with mortality rising with the age of the population and burden of associated comorbidities [41]. However, as standards of care increase, mortality in younger age groups may continue to decline. The emerging resistance of pneumonia-related pathogens to commonly used antibiotics has been documented across Europe, although correlation with mortality could not be established in a number of these studies [6]. These resistant pathogens may lead to increasing treatment failure and could have a significant impact on the future of pneumonia-related mortality. The development of international guidelines for antibiotic use, the development of new therapeutic interventions, and the implication of effective vaccination programmes may all influence the future burden of pneumonia in Europe.

The main strength of this study is the overall totality of data analysed. Specifically, we have utilised national mortality statistics for a significant number of countries over a long observation period. The data which is deposited into the database is reported and validated at the national level and therefore removes the possibility for sampling error. We have included a high number of countries across the European region in order to provide comparisons between health systems. Our decision to analyse trends over time within each country also helps to minimise year-to-year variations in favour of overall trend analysis. The monitoring and analysis of national mortality statistics is valuable to assess various economic, policy and health-related interventions. The results of this investigation could, for example, help policy and decision-makers to identify strategies to successfully reduce the burden of pneumonia on mortality by comparing interventions and their effects across various health systems. The mortality data used in this study has been collected over a 14-year period and was obtained from a heterogeneous cohort of countries across the WHO European Region. Our study is limited by the accuracy of the certification of death and disease classification across a vast number of different countries. Potential variability in death certification and coding of mortality may limit interpretation of absolute mortality rates between nations. We focused on changes in mortality rates over time and mortality trends for interpretation. There is a paucity of evidence comparing death certification for pneumonia with true cause of death, with one study suggesting substantial discrepancy [42]. Furthermore, it may be reasonable to assume that accuracy of coding practices may vary over time as new revisions of the ICD coding classification are implemented. To facilitate the effective utilisation of its data, the WHO reviews its mortality data for accuracy in reporting and satisfactory coverage. A limitation of use of the ICD-10 classification is the potential underestimation of the pneumonia mortality burden by not including pneumonias as an immediate or contributory cause of death. This disadvantage has been identified in a number of studies looking at the mortality burden of pneumonia [32, 43]. One further potential limitation is the influence of missing data. We chose to exclude from our analysis countries with a significant (i.e. > 20%) proportion of missing data and imputed where countries had a smaller proportion of missing data. Exclusion of these nine EU counties may reduce the generalizability of our findings. In total, 4% of data were imputed and it is possible, although unlikely, that these may significantly influence the results.

## Conclusion

Our study highlights a general decline in pneumonia mortality in the European area over the period 2001 to 2014. Important discrepancies between genders and geographical regions were also identified, the reasons for which are likely multi-factorial and may result from the interplay between medical, social, economic and environmental factors.

## Additional file

Additional file 1: **Figure S1.** Pneumonia mortality trends of male and females in 19 European countries including influenza-related mortality shown in red overlay. Lines represent result of Joinpoint analyses: dashed and continuous lines for males and females, respectively. (DOCX 401 kb)
