# Supplementary material for: Trends in mortality from pneumonia in the Europe union: a temporal analysis of the European detailed mortality database between 2001 and 2014
Source: Respir Res. 2018 May 4;19:81. doi: 10.1186/s12931-018-0781-4 (PMC5935998; doi:10.1186/s12931-018-0781-4)
Supplement: Supplementary file 1 — Figure S1. Pneumonia mortality trends of male and females in 19 European countries including influenza-related mortality shown in red overlay. Lines represent result of Joinpoint analyses: dashed and continuous lines for males and females, respectively. (DOCX 401 kb) [file 12931_2018_781_MOESM1_ESM.docx]

**Additional file 1**

**Figure S1:** Pneumonia mortality trends of male and females in 19 European countries including influenza-related mortality shown in red overlay. Lines represent result of Joinpoint analyses: dashed and continuous lines for males and females, respectively. Squares (males) and circles (females) represent raw data.
